# Does Subjective Rating Reflect Behavioural Coding? Personality in 2 Month-Old Dog Puppies: An Open-Field Test and Adjective-Based Questionnaire

**DOI:** 10.1371/journal.pone.0149831

**Published:** 2016-03-15

**Authors:** Shanis Barnard, Sarah Marshall-Pescini, Chiara Passalacqua, Valentina Beghelli, Alexa Capra, Simona Normando, Annalisa Pelosi, Paola Valsecchi

**Affiliations:** 1 Dipartimento di Neuroscienze, Università degli Studi di Parma, Parma, Italy; 2 Comparative Cognition, Messerli Research Institute, University of Veterinary Medicine, Vienna, Medical University of Vienna, University of Vienna, Vienna, Austria; 3 Wolf Science Centre, Ernstbrunn, Austria; 4 Sez. di Neuroscienze, Dipartimento di Fisiopatologia medico-chirurgica e dei Trapianti, Università di Milano, Milan, Italy; 5 Dipartimento di Scienze Mediche Veterinarie, Università degli Studi di Bologna, Bologna, Italy; 6 Gentle Team, Associazione Sportiva Dilettantistica, Asti, Italy; 7 Dipartimento di Biomedicina Comparata e Alimentazione, Università degli Studi di Padova, Padua, Italy; Liverpool John Moores University, UNITED KINGDOM

## Abstract

A number of studies have recently investigated personality traits in non-human species, with the dog gaining popularity as a subject species for research in this area. Recent research has shown the consistency of personality traits across both context and time for adult dogs, both when using questionnaire based methods of investigation and behavioural analyses of the dogs’ behaviour. However, only a few studies have assessed the correspondence between these two methods, with results varying considerably across studies. Furthermore, most studies have focused on adult dogs, despite the fact that an understanding of personality traits in young puppies may be important for research focusing on the genetic basis of personality traits. In the current study, we sought to evaluate the correspondence between a questionnaire based method and the in depth analyses of the behaviour of 2-month old puppies in an open-field test in which a number of both social and non-social stimuli were presented to the subjects. We further evaluated consistency of traits over time by re-testing a subset of puppies. The correspondence between methods was high and test- retest consistency (for the main trait) was also good using both evaluation methods. Results showed clear factors referring to the two main personality traits ‘extroversion,’ (i.e. the enthusiastic, exuberant approach to the stimuli) and ‘neuroticism,’ (i.e. the more cautious and fearful approach to the stimuli), potentially similar to the shyness-boldness dimension found in previous studies. Furthermore, both methods identified an ‘amicability’ dimension, expressing the positive interactions the pups directed at the humans stranger, and a ‘reservedness’ dimension which identified pups who largely chose not to interact with the stimuli, and were defined as quiet and not nosey in the questionnaire.

## Introduction

In recent years there has been an increasing interest in the evolutionary significance of the concept of personality, making it an appealing research topic for comparative psychologists, biologists, and evolutionary scientists alike. As a consequence, many species have become the object of interest for personality-researchers [1], and one species that has received increasing attention is the domestic dog (*Canis familiaris*) [2–4]. The reasons for this interest are varied, but the potential applicability, for example in helping to find appropriate homes for shelter dogs [5–7], selecting the most appropriate puppies for working dog training [8–14], and the early detection of behavioural problems that may hence obtain rapid treatment [15,16], has had a strong impact on the field.

Various methods have been used to assess the personality of dogs. Behavioural testing has been adopted in a number of studies using a variety of tests [17–19]. This method involves presenting a selection of stimuli/situations to dogs in a standardized manner, to allow comparisons to be made. Behavioural coding can then be combined either with detailed analyses of behaviour (mostly done at a later stage from video) or using rater coding of behavioural categories (which can be done either *in situ*, from video, or both). Detailed analyses of behaviour normally involves measuring frequency, duration and latency of specific behaviours, for example coding the occurrence of behaviours such as bared teeth, raised hackles, and growling [20,21]. Rater-coding is normally done on a predetermined scale [22], which can measure either the presence/absence of a particular behaviour/behaviours (e.g. whether the dogs do or do not exhibit stress signals, which would then include, lip-licking, yawning, etc.) or the intensity of its exhibition (e.g. how much a dog rates on a scale of 1 to 5/7 on for example aggression, each score would then coincide with the presence/absence of a certain combination of behaviours relating to aggression such as growling, baring teeth, snapping etc.) [20].

Finally, numerous studies have used a questionnaire-based approach, where typically either the owner or a person well acquainted with the dog rates the dog’s behaviour in everyday situations [23]. In a number of cases, the questionnaire was validated using a behavioural test or other measure (vet visit in the case of behavioural problems for example) by comparing results obtained with the two different methods [21,24–26]. However, only a few attempts have been made to evaluate the degree to which proposed canine personality dimensions predict observed behaviour of individuals in contexts different from those in which they were developed [25–27].

Each method used independently has its pros and cons. The problem with behavioural testing is that, aside from the time-consuming aspect of carrying out the test (which can last anything between 2 to 30 minutes), whether using rater-coding, or an in depth analyses of specific behaviours, it requires evaluators to be trained and knowledgeable of dog behaviour, and in the latter case, it requires at least the same amount of time in coding than it did in testing. Questionnaire-based evaluations are much faster, however, so far they have mostly been used with dogs being exposed to a number of different situations (repeatedly), or by asking owners to assess the dogs’ behaviour across many contexts. Furthermore, questionnaire-based evaluations are considered to be more subjective, since the assessment of the dog’s behaviour is filtered through the person’s perception of the dog (including long-held, but perhaps no longer applicable beliefs, or breed prejudices etc.), although according to some authors, given they are normally based on a much wider perspective and information base, they may prove more accurate [28].

A recent meta-analysis of data from 31 studies determined that there is moderately high temporal consistency (R = 0.43) in dog personality scores, with no differences in consistency between personality scores based on behavioural ratings versus behavioural coding [3]. Given the different methods are in theory measuring the same underlying trait, it is surprising how few studies have simultaneously used different evaluation tools. Perhaps, even more worryingly, where questionnaires and behavioural tests have been used in the same study, correlation coefficients among similar personality traits are usually low, falling between 0.2–0.4 [26,29,30]. When questionnaire and behaviour coding targeted very specific traits, then higher correlations emerged. For example, Kubinyi et al.[31] found that dogs assessed by owners as more active-impulsive and inattentive showed also more activity in four behaviour test situations (r = 0.53 and r = 0.25, respectively).

Overall, there is a fair amount of agreement in regards to the personality factors emerging from the behavioural and questionnaire data. However, most factors have been extrapolated from studies assessing the behaviour of adult pet dogs. Hence, it is not clear whether an evaluation of puppy personality (behavioural or questionnaire-based) would result in the same factors emerging in adult dogs. Furthermore, whereas a few adult-based studies have sought to establish the correspondence between behavioural testing and questionnaire-based evaluations [26,29,32,33], this has not been the case for studies with young puppies, where, to our knowledge, only behavioural testing with coder rating has been used to assess personality [34].

The aim of the current study was therefore to use an open-field test accompanied by a simple adjective-based questionnaire to test personality traits of 2-month-old dog puppies and assess the correspondence between these two methods of evaluation. To achieve our aim, a sample of 2-month old puppies was tested at their breeders. Independent observers carried out behavioural analyses from videos of the pups in an open-field test, and a different set of independent observers (unaware of the behavioural coding done by the others) scored each pup on a previously selected adjective-based questionnaire. The *correspondence* between the two methods was assessed by comparing the personality factors emerging from the results of the behavioural analyses with those emerging from the questionnaires-based evaluation. *Consensus* (i.e. inter-observer reliability) for the behavioural analyses in the open field test and the adjective-based questionnaire was assessed as a pre-requisite prior to all analysis. To further investigate the validity of the personality assessment using these different tools we assessed the *internal consistency* of the personality dimensions in the adjective-based questionnaire (i.e. the degree to which judgments about an individual’s personality are consistent across items (i.e. adjectives) thought to reflect the same behavioural dimension [35,36]. Finally, *trait consistency* (test-retest reliability) was assessed by re-testing a sample of puppies two months later with the same test and comparing results both in terms of the behavioural analyses and questionnaire-based evaluation.

At a theoretical level, identifying personality factors at an early age may increase the likelihood that these dimensions have a genetic component (i.e. endophenotyping), hence potentially providing a tool for gene-behaviour studies and adding to the growing interest on the evolutionary perspectives of personality [37]. Furthermore, a more general tool to assess personality in young puppies may have important outcomes also in a more applied setting, since personality has been shown to be an important variable in determining owner satisfaction [38].

## Methods

### Ethics statement

No special permission for use of animals (dogs) in such behaviour studies is required in Italy, however when first visiting the breeders, an in depth description of the test was presented by the researcher and consent, to video-record and use data in an anonymous form, was sought verbally prior to testing. Following agreement they compiled a form with details regarding the dog breeding activity (e.g. type of breed, number of females, number of litters per year etc.). Since breeders did not have an active role in the study and were never subjected to evaluation themselves, IRB or equivalent was not required. All procedures were performed in full accordance with Italian legal regulations and the guidelines for the treatments of animals in behavioural research and teaching of the Association for the Study of Animal Behavior (ASAB).

### Subjects

A total of 79 litters, representing 21 breeds (range: 1 to 10 litters per breed, median: 3 litters per breed; mean: 3.8 litters; Table 1), were included in the study. Litters came from a total of 55 registered dog breeders. Wherever possible, we chose more than one breeder for each breed to avoid the risk of testing specific bloodlines. All puppies were tested at the breeders before adoption.

**Table 1:** Summary of the analysis carried out and details on sample size, breed, sex (M, F), number of litters from which the sample was taken associated to each analysis.

Assessment	Sample	M	F	Breeds (litters)
Selection of questionnaire (S1 Information)	15	8	7	Alaskan Malamute (1), American Pit Bull (1), American Staffordshire (1), Argentinian Dogo (1), Australian Shepherd (1), Border Collie (1), Boxer (1), Czecholslovakian Wolfdog (1), Doberman (1), English Bull Terrier (1), German Shepherd (1), Golden Retriever (1), Labrador Retriever (1), Rottweiler (1), Siberian Husky (1)
(1) Hierarchical Cluster analysis of behavioural data; (2) Internal consistency of questionnaire personality dimension (3) Confirmatory Factor analysis of questionnaire data	154	79	75	Akita Inu (1), Alaskan malamute (2), American Pit Bull (2), American Staffordshire (7), Argentinian Dogo (5), Australian Shepherd (6), Bolognese (1), Border Collie (4), Boxer (10), Cavalier King Charles (1), Czechoslovakian Wolfdog (1), Doberman (3), Drahthaar (1), English Bull Terrier (1), French Bulldog (1), German Shepherd (7), Golden Retriever (8), Labrador Retriever (8), Rottweiler (5), Siberian Husky (4), West Highland White Terrier (1)
Inter-observer agreement for behavioural coding (Consensus analysis)	[63]	[32]	[31]	
Inter-observer agreement for questionnaire coding (Consensus analysis)	[60]	[30]	[30]	
Trait consistency (test-retest reliability) between pups at 2 and 4 months old	18	9	9	American Pit Bull (1), Australian Shepherd (2), Boxers (2), Labrador Retriever (1)

Numbers in [] are a sub-sample of the total 154 puppies.

An initial sample of 15 video-recorded tests, chosen to represent as much as possible the variability of the whole subject pool, was used only for the adjective-based questionnaire selection process (S1 Information). In this sample pups were balanced for sex (8 M; 7 F) and were representative of 15 different breeds (Table 1).

A sample of 154 puppies (79 males and 75 females) tested at 2 months (range 58–62 days) was selected from the above mentioned 79 litters taking a maximum of 2 puppies (1 male and 1 female wherever possible) from each litter (Table 1). The video coding of the tests carried out with this sample were analysed using both the behavioural and questionnaire-based method to allow an analysis of the personality traits emerging from the two different methods and potential correspondence between them.

Finally, in order to assess the trait consistency (test-retest reliability), a sample of eighteen puppies was tested at both 2 and 4 months of age. This was possible because these pups were not sold but kept by the breeders. Therefore, the test, the environment and the owner were exactly the same as when they were first tested. The questionnaire evaluation was also carried out on these subjects tested at different ages (see Table 1, for subject sex and breed).

### Procedures

#### Open field test

**Fig 1:**
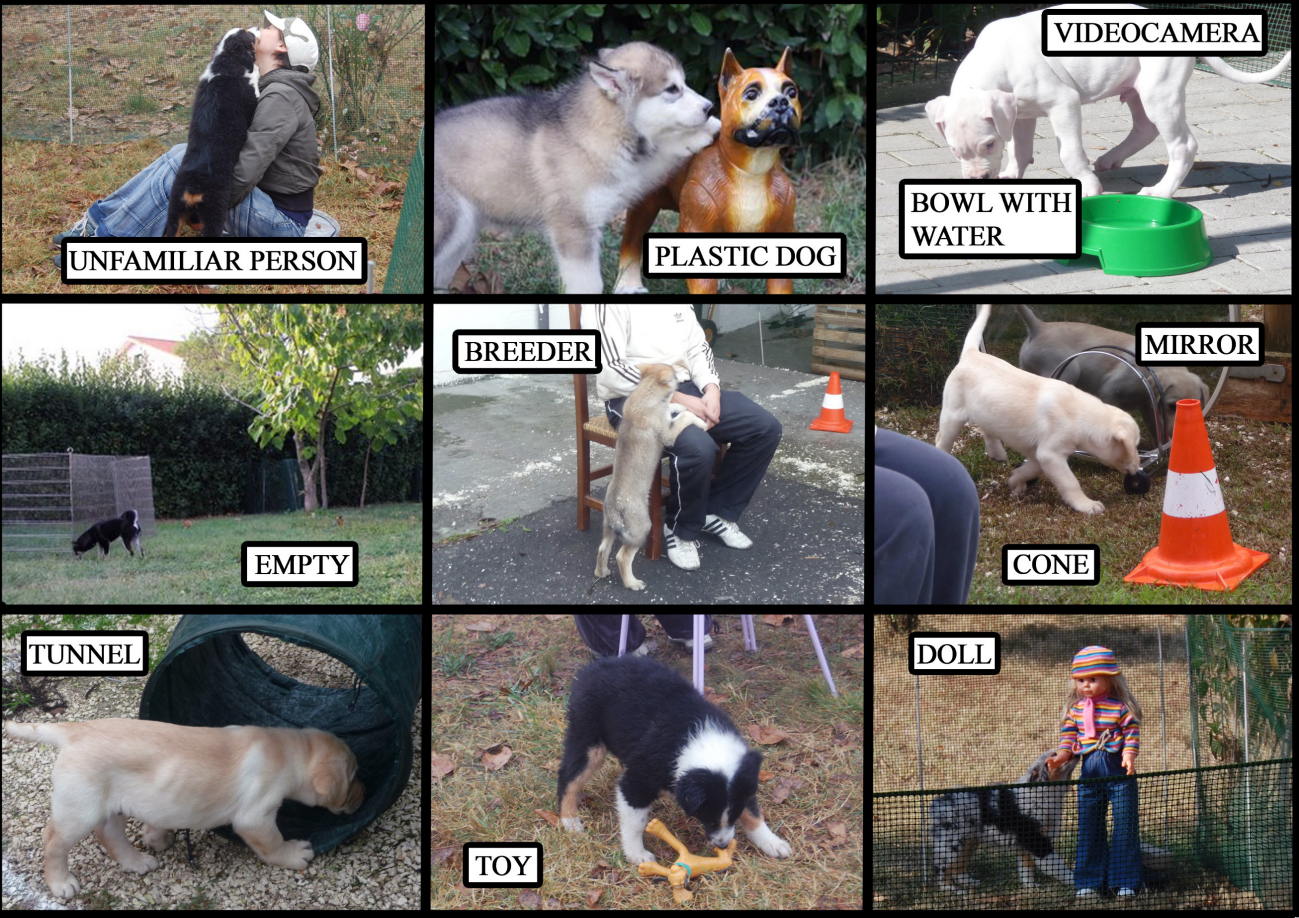
Stimuli and setup in the modified open-field test.

The open field test was carried out at the breeder’s place in a quiet, 5 x 5 m area, temporarily fenced off, using a portable ‘puppy pen’ (1m high) covered by a dimming net (to avoid distraction from the outside). Testing was normally carried out in the morning (9-11h), but could vary according to the breeder availability. Using powdered chalk, the area inside the pen was divided into 9 identical squares (Fig 1).

Each square contained a stimulus: 1) a realistic looking plastic dog (approx. 50 cm tall), displaying a rather assertive, erect posture, ears forward and docked tail, 2) a bowl of water, 3) a street cone, 4) a mirror propped up to be at puppy height, 5) a child-looking doll standing up (approx. 86 cm high) and positioned with her arms reaching in front of her, and her upper body slightly bent forward; 6) a squeaky dog toy; 7) a small nylon tunnel (53 cm long and 43 cm diameter), similar to those used in agility, with a small piece of food placed inside it. One square was left empty. Finally, two people were also present in the pen: the breeder, seated on a chair in the centre square, and a female researcher, seated on the ground in a corner square. The breeder was asked not to interact with the puppy and remain passive during the test. The experimenter seated on the ground, behaved in a natural way with the pup, in that she did not call or invite interaction but if the pup engaged with her she would briefly respond by petting it then would stop interacting and adopt a relaxed posture. The position of the stimuli was the same for all pups tested.

The breeder was asked to carry the pup into the pen, and once seated, place the pup on the ground in front of his/her feet. The pup was then free to move around in the pen for 5 minutes. A video camera was set up on a tripod outside the pen, and manoeuvred by an assistant so as to insure the pup’s behaviour was recorded during the whole test.

#### Selection of an Adjective-based Questionnaire

All questionnaires concerning the personality of dogs available in the literature, which have been specifically designed for use by owners, and mostly refer to the everyday situations dogs may be observed in, were excluded since they are difficult to apply to our current setting. A sub-sample of owner-directed questionnaires however, which were concerned more with scoring the dog’s personality on a number of characteristics best described by adjectives, were used. Of course the owners still refer to their knowledge of the dog in a daily context; however, adjective-based questionnaires, being less specific of the context, may be more easily applied to our research. Initially, four potential adjective-based questionnaires were taken into consideration: the canine Big Five Inventory (BFI) [26]; the Monash Canine Personality Questionnaire (MCPQ/MCPQ-Refined) [39–41]; the demographic personality questionnaire [2] and the Free-choice profiling method [42]. However after a systematic selection process (see S1 Information), a modified version of the Ley et al. [40] was used (Table 2).

**Table 2:** List of adjectives in the five personality subscales derived from the Ley et al. [37] and those used in the current study.

Dimension	Adjectives
Extraversion	**Active**, **eager**, **energetic**, **enthusiastic**, **excitable**, **exuberant**, **hyperactive**, **lively**, **quiet**, **restless**
Neuroticism	**Cautious**, **fearful**, **nervous**, **sensitive**, **timid**, submissive,
Amicability	**Easy-going**, **friendly**, **gentle, happy-go-lucky**, **sociable**, non-aggressive, **relaxed**, unaggressive
Self-assuredness/Motivation	**Assertive**, **determined**, **independent**, **nosey**, **persevering**, **tenacious,** dominant, opportunistic, proud, thorough
Training Focus	Attentive, biddable, intelligent, obedient, reliable, trainable, clever

**Adjectives in bold**: adjectives retained in the present study.

### Coding and Analyses

The authors of this paper carried out all video analyses, for both behavioural and questionnaire coding.

#### Behavioural Coding and Analyses

After viewing approximately 30% of randomly chosen tests, an ethogram of the puppies’ behaviour during the open field test was determined (Table 3).

**Table 3:** Behavioural variables recorded during the study.

Label	Behavioural Description
**Walk**	To move along on foot, advancing step by step whilst looking around, or looking outside the enclosure but at no object/stimulus in particular.
**Fast gait**	To move either trotting, cantering or galloping/bounding whilst looking around, or looking outside the enclosure but at no object/stimulus in particular.
**Cautious approach/interaction (object or people)**	*Risk assessment*: The dog starts off by keeping its body at a distance from the object and extending only its upper body towards it. It looks like the dog is stretching towards the object. This is often accompanied by hesitant and jerky back and forth movements.
** **	*Olfactory inspection with lowered posture*: sniffing of the object displaying slow movements, with ears and tail held low and potentially also back legs bent.
**Positive approach/interaction (object or people)**	The pup approaches the stimulus in a direct manner, sniffs it with tail hanging, held parallel or slightly above the bodyline. The tail may be still or slow wagging. The mouth is relaxed and the ears are pricked forward. The pup may lick or touch the stimulus with its paws. If towards the ‘tunnel’ the pup explores also the interior, moving inside it with at least the front paws.
**Exuberant approach/interaction (object or people)**	The pup approaches the stimulus at a fast walk, trot, run or bounding towards it. Often it throws the object over with the impetus of its movements. It sniffs the object with tail held higher than the line of the body, wagging it rapidly, never stopping in one place but sniffing the object all over whilst moving continuously. The mouth is relaxed, the ears pricked forward. The body posture is tall. It may lick and touch the object with its paws. If directed towards the ‘tunnel’ it may run through it.
**Social interaction (only if referring to the experimenter or the breeder)**	*Greeting*: to interact in a friendly manner, holding the ears back, with a relaxed open mouth, the tail held low and wagging rapidly especially with the end part of the tail, occasionally accompanied by whining. Pups may also lick, sniff or gently prod the persons’ face or mouth.
** **	*Hurtle*: standing on back legs often associated with jumping up towards the person’s face whilst exhibiting a fast and wide tail wagging motion. With an increase in the excitement level, the jumping up behaviour becomes more intense and it may be accompanied by muzzle hits or biting of the person’s clothes, hair, face and hands.
** **	*Lap-sitting*: the pup climbs into the experimenter’s lap, sitting or lying on her knees.
** **	*Belly up*: the pup lies down next to the person, displaying its belly.
** **	*Contact-seeking*: extending a paw to touch the person, or using the nose/snout touching the person or placing it on the person’s lap.
**Playful interaction (object or people)**	*Play bow*: to lower the front part of the torso while keeping the hind part upright.
** **	*Play-related behaviours*: A display of a number of predatory type behaviours such as grabbing, holding an object in the mouth and head-shaking, mouse jumping on an object, pulling and biting but changing position frequently, all displayed in a non-aggressive manner.
**Carry toy**	Holding the toy (usually the squeaky toy) in the mouth whilst walking, trotting or running around in the arena. If however, the exaggerated behaviours typical of play were exhibited whilst carrying the toy, we coded the behaviour within the ‘playful interaction’ category and not the ‘carry toy’ one.
**Deflection (object or people)**	*Avoidance*: after looking intently at the stimulus the pup first looks away then changes the orientation of its body in a direction opposite to the stimulus’ location or maintains a position so as not to shorten the distance between itself and the stimulus. This is however usually accompanied by rapid glances to the ‘offending’ object.
** **	*Startle response*: a sudden movement in the opposite direction to the ‘alarming’ stimulus, whilst maintaining the head oriented towards it.
** **	*Walk backwards*: the pup increases the distance between itself and the stimulus whilst maintaining the body oriented towards it.
**Look at stimulus (object or people)**	Visual exploration of the stimulus, the dog is oriented and looking towards it from at least a few paces away. This behaviour often occurs just before an interaction or avoidance of the object. If the pup is looking at the stimulus and walking parallel to it, the ‘look at stimulus’ behaviour ‘over-rides’ the walk/trot category outlined above.
**Non-stimuli-related behaviour**	This category captures the time pups spent not interacting/engaging with the stimuli. The pup is either in a static position (sitting, lying or standing), perhaps looking around (e.g. outside the fence but not towards the stimuli) and/or biting chewing on elements of the surrounding environment such as grass, sticks, leaves etc. or is moving within the field whilst sniffing at the ground or at the fence
** **	*Maintenance behaviours* i.e. drink, eat biscuit (which was in the tunnel), elimination behaviours

Because the aim of the current study was to assess whether there are broad personality traits already visible in 2 month old puppies, we chose a midlevel analysis, i.e. rather than focus on single units of behaviours such as, the frequency of tail wags or the number of startle responses etc., we opted for a more global assessment of the dogs’ behaviour, especially those behaviours directed towards the stimuli presented. However, although the approach to the single stimulus was recorded as cautious, relaxed or exuberant, the assessment of which category to include the pup in was based on an objective observation of the pups’ behaviour in terms of body posture, speed of approach and tail movement (see Table 3 for a detailed description of these). Deflection from the stimuli (including avoidance behaviours, moving back/away, or a startle response followed by a change of direction) was also coded, as well as social and play behaviours directed either at the people or objects. These midlevel interpretations of the dogs’ behaviour were defined on the basis of specific behavioural patterns emerging from the literature (see Table 3) [43–45]. In total 11 mutually-exclusive behavioural categories were recorded continuously in terms of duration of their occurrence. The occurrence of these behaviours was scored as directed towards each stimulus in the test enclosure. Video analyses of behaviours were carried out using behavioural event recording software (Observer XT 8.0, Noldus Information Technology, The Netherlands).

Two observers (SB and VB) scored 154 videos and 40% of these were coded by both. Consensus (inter-observer agreement) was evaluated using Cronbach’s alpha. A preliminary Principal Component Analysis was carried out to identify main clusters of behaviours but the KMO (Kaiser-Meyer-Olkin measure of sampling adequacy) was too low (0.539) to validate the analysis hence we decided to collect behaviours by means of a hierarchical cluster analysis (method: average—linkage between groups; similarity measure: Euclidean squared distance; (see e.g. [46,47]) using only behaviours shown by at least 30% of subjects (all behaviours reported in Table 3 except carry toy, deflection and other). The hierarchical cluster analysis creates subsets (or clusters) of objects (i.e., observations, individuals, items of variables) such that those within each cluster have a higher degree of similarity than objects assigned to different clusters. Similarities (or dissimilarities are defined by an appropriate metric (a measure of distance between pairs of observations), and a linkage criterion.

To evaluate trait consistency (test-retest reliability), the videos of the 18 pups tested both at 2 and 4 months of age were all coded by the same observer (SB) and a Spearman’s correlation on the main behavioural clusters was computed.

#### Questionnaire Coding and Analyses

All 154 puppies’ tests were coded by SMP (unaware of the ethogram used in the behavioural analyses) using the adapted version of the Ley et al. [40] questionnaire.

A second coder (SN; also unaware of the ethogram used in the behavioural analyses) used the questionnaire to score a random selection of puppy tests (39% of the total). These data were used for consensus (inter-observer reliability) analyses using Cronbach’s alpha. For inter-observer coding, each adjective was analysed independently to evaluate which adjectives received a greater or lesser consensus when evaluating puppy behaviour.

Maintaining, the same behavioural dimensions identified by Ley et al. [40], the internal consistency of the test was calculated using Cronbach’s alpha (the average value of the reliability coefficients one would obtained for all possible combination of items when split into two half-tests) and the mean inter-item correlations (providing an assessment of item redundancy and representativeness of the content domain). It is reported that the combination of both this scores is the most accurate measure of internal consistency [48].

Cronbach’s alpha values expressing the internal consistency on the personality dimensions (see results section) were comparable to those reported for adult dogs by Ley et al. (which varied from 0.74 to 0.87) [40]. However, inter-item correlations were in some cases substantially low. Hence, since no study has been carried out using a questionnaire based methodology on puppies, we ran a Confirmative Factor Analyses (CFA using the Maximum Likelihood extraction method as rotation method, and setting the factor loading at 0.40 [49,50]) to assess whether the adjectives identified by Ley et al. [40] for adult dogs would group into similar factors also in puppies. Finally, Pearson’s correlation coefficients among the personality factors were evaluated.

Finally, SMP also scored all the puppies tested at 2 and 4 months and consistency (test-retest reliability) in scoring at these two time intervals was assessed using Spearman’s correlation coefficient.

#### Correspondence between behavioural and questionnaire-based method

Correspondence refers to the extent to which judgments predict an external criterion for “reality” [51]; previous studies identified independent observations of behaviours as the most valuable external criterion [52,53]. Bivariate linear correlations were carried out using Pearson’s r between each personality trait emerging from the questionnaire-based factorial analyses and behavioural clusters emerging from the cluster analysis.

## Results

### Behavioural Analyses

#### Consensus

Inter-observer reliability between the two observers showed an average Cronbach’s alpha of 0.87 in behavioural scoring. In particular, the Cronbach’s alpha carried out on duration of main behavioural categories was as follows: cautious approach/interaction = 0.58, playful interaction = 0.97, walk = 0.97, deflection = 0.87, fast gait = 0.95, carry toy = 0.97, social interaction = 0.77, look at stimulus = 0.96, exuberant approach/interaction = 0.93, positive approach/interaction = 0.98, non-stimuli related behaviour = 0.97.

#### Hierarchical cluster analysis

**Fig 2:**
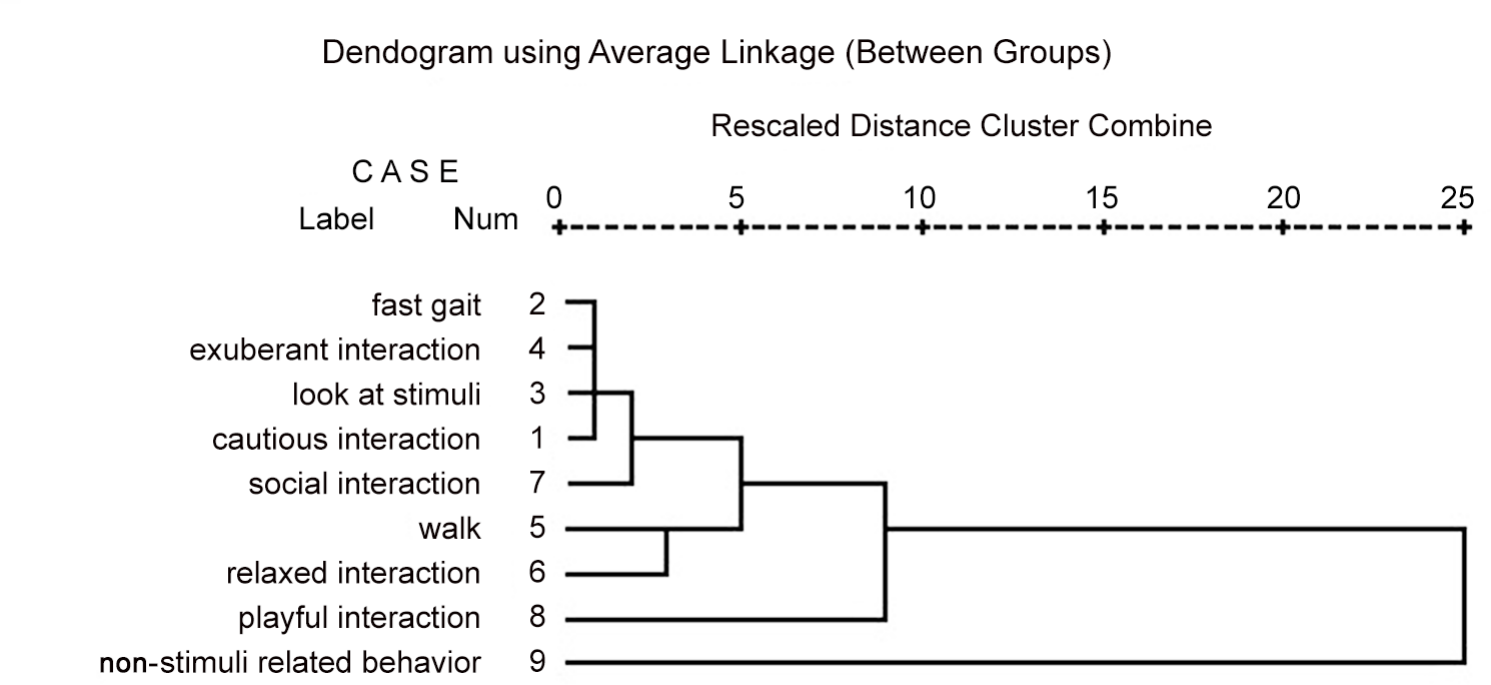
Behaviours hierarchical cluster analysis: agglomeration dendrogram.

The dendrogram visual inspections along with the agglomeration matrix of the cluster analysis (maximum increment between stadiums criterion) suggested a 5 clusters solution (Fig 2): at the first stadium, we found a cluster composed by two subsets of symmetrical behaviours (exuberant approach/interaction and fast gait labelled “exuberant attitude” vs. look at stimuli and cautious approach/interaction labelled “cautious attitude”). The first subset outlines puppies behaving in one of two extreme manners: either hurtling towards all stimuli with boundless enthusiasm, the second outlines puppies looking at the stimuli from afar and when choosing to interact with them, doing so with a measure of anxiety and caution.

At the third stadium a second cluster containing walk and positive approach/interaction, named “relaxed attitude” emerged. This cluster described those puppies that were relaxed in their interaction, investigating the objects in a positive way, yet without showing the extreme exuberance of those pups included in the ‘exuberant’ attitude group.

Social interaction converged with the first cluster at the fourth stadium; last, playful interaction and non-stimuli related behaviour did not converge and remained as single items until the end of the agglomeration program.

#### Trait consistency (test-retest reliability)

Spearman Rho correlation tests were carried out on the behavioural clusters of puppies tested at 2 months of age and retested at 4 months of age. Significant correlations emerged for two clusters: exuberant attitude (r = 0.69, p = 0.002) and cautious attitude (r = 0.50, p = 0.04) [relaxed attitude (r = 0.26, p = 0.29), social interaction (r = -0.23, p = 0.36), playful interaction (r = 0.42, p = 0.08), non-stimuli related behaviour (r = -0.20, p = 0.94).

### Questionnaire Analyses

#### Consensus

Inter-observer reliability between the two observers based on 40% of the tests showed a Cronbach’s alpha of 0.87. Cronbach’s alpha expressing the consensus for single adjectives varied from 0.52 to 0.90 (Table 4).

**Table 4:** Inter-observer reliability measures for each adjective used to assess puppies.

Adjectve	alpha	Adjectve	alpha
Active	0.83	Happy go lucky	0.65
Assertive	0.72	Indepedent	0.72
Cautious	0.85	Lively	0.81
Determined	0.67	Nervous	0.70
Eager	0.80	Nosey	0.66
Easy going	0.70	Persevering	0.59
Energetic	0.86	Quiet	0.76
Enthusiastic	0.90	Relaxed	0.49
Excitable	0.67	Restless	0.52
Exuberant	0.89	Sensitive	0.57
Fearful	0.67	Sociable	0.79
Friendly	0.81	Tenacious	0.67
Gentle	0.61	Timid	0.83

#### Internal consistency and Factor reduction

Mean *internal consistency* on the four personality dimensions identified by Ley et al. [40] was calculated on the data from the whole subject pool (154 puppies), coded by a single observer (SMP). Cronbach’s alpha (and inter-item correlation scores) was: 0.87 (0.45–0.94) for extraversion; 0.87 (27–82) for neuroticism, 0.80 (0.004–0.92) for amicability, and 0.77 (0.09–0.70) for self-assuredness.

The Confirmative Factor Analysis (Maximun Likelihood, orthogonal Varimax rotation, based on adjectives in Table 5) did not confirm the goodness of fit of a four-factors model (χ^2^ = 540.3, df = 227, p < .01; RMSEA = 0.091; CFI = 0.92; 21% not redundant residuals >0.05, [54]), but highlighted that the better, even optimal, model was a 5 factors solution accounting for 76.95% of the variance with substantial, but not complete, overlap with factors identified by the Ley study (χ ^2^ = 414.23, df = 226, p < .01; RMSEA = 0.07; CFI = 0.95; 12% not redundant residuals >0.05 [54]). The five factors were labelled: Extraversion, Neuroticism, Persistence Amicability, and Reservedness (Table 5).

**Table 5:** Factor loadings from the questionnaire-based confirmative factorial analysis. Factor loadings > 0.40 are in bold.

	Factors				
Adjectives	1 Extraversion	2 Neuroticism	3 Persistence	4 Amicability	5 Reservedness
Enthusiastic	**0.897**	0.273	0.183	0.121	0.140
Exuberant	**0.887**	0.188	0.196	0.136	0.205
Happy-go-lucky*	**0.839**	0.267	0.124	0.244	-0.064
Energetic	**0.836**	0.269	0.242	0.148	0.196
Lively	**0.836**	0.168	0.220	0.166	0.232
Excitable	**0.831**	0.209	0.266	0.094	0.131
Eager	**0.790**	0.271	0.243	0.183	0.105
Active	**0.712**	0.280	0.227	0.203	0.272
Easy-going*	**0.608**	0.563	0.136	0.112	-0.029
Hyperactive	**0.426**	0.041	0.413	0.164	-0.003
Nervous	-0.215	**-0.838**	-0.082	0.018	-0.106
Fearful	-0.265	**-0.819**	-0.120	-0.080	-0.133
Cautious	-0.237	**-0.756**	-0.216	-0.189	-0.207
Relaxed*	0.281	**0.709**	-0.148	0.113	0.019
Timid	-0.511	**-0.521**	-0.285	-0.136	-0.483
Independent*	-0.004	**0.519**	0.154	0.031	0.040
Sensitive	-0.268	**-0.449**	-0.329	-0.003	-0.325
Persevering*	0.115	0.055	**0.763**	-0.021	-0.144
Tenacious*	0.370	0.334	**0.696**	0.099	-0.050
Determined*	0.268	0.318	**0.663**	0.203	0.098
Gentle*	-0.038	0.052	**-0.583**	0.091	-0.258
Restless*	0.480	0.014	**0.530**	0.066	0.083
Assertive*	0.368	0.292	**0.483**	0.123	0.182
Sociable*	0.545	0.157	0.050	**0.804**	0.145
Friendly*	0.572	0.204	0.124	**0.682**	0.157
Quiet*	-0.561	-0.239	-0.265	-0.124	**-0.605**
Nosey*	0.187	0.346	-0.093	0.174	**0.465**
**Variance explained**	**50.87**	**9.78**	**8.27**	**4.32**	**3.71**

* Indicates adjectives grouping differently in the current study compared to Ley et al. [40].

Pearson’s correlation matrix showed that the first factor Extraversion was strongly and positively related to Persistence (r = 0.606) and to Amicability (r = 0.739) and was negatively associated to Neuroticism (r = -0.622) and to Reservedness (r = -.639). A higher score on Neuroticism was related to a weaker value in Persistence (r = -0.512) and Amicability (r = -0.463) and to a higher Reservedness (r = 0.650). Persistence was also related to Reservedness (r = -0.412) and Amicability (r = 0.391); the latter was negatively correlated to Reservedness (r = -0.554).

Hence, the general picture emerging suggests that subjective rating using an adjective based questionnaire is capable of picking out five specific types of puppies. The two major traits (Extraversion and Neuroticism) described respectively either an energetic puppy, who bounded towards the stimuli and explored them whilst displaying a relaxed posture and much tail-wagging, or conversely, a puppy that tended to either avoid the stimuli looking at it from afar, spending time sniffing around and interacting with other elements of the environment or approached them but with a slow gait, in a tentative manner, holding the tail and potentially the hind quarters low, and hence exhibiting signals of mild fear or apprehension.

The Reservedness trait identified those pups whom did not show particular fear or unease in relation to the stimuli but largely chose not to interact with them. The questionnaire also successfully identified the puppies that interacted in a positive manner with the stranger (amicability). The persistence trait was somewhat more problematic, correlating with too many behavioural factors, to allow a clear characterization of exactly what type of puppy it described.

#### Trait consistency (Test-retest reliability)

Spearman Rho correlation tests were carried out on questionnaire evaluation of puppies at 2 months of age retested at 4 months of age. A significant correlation, between scores at 2 and 4 months emerged only for three factors i.e. Extraversion (r = 0.80, p<0.001), Neuroticism (negative loadings: nervous, fearful, cautious, timid, sensitive; r = 0.67, p = 0.003) and Reservedness (negative loading: quiet; r = 0.56, p = 0.02); [non-significant results: Neuroticism (positive loadings: relaxed, independent; r = 0.35, p = 0.17); Persistence (negative loadings: gentle r = 0.14, p = 0.61); Persistence (positive loadings: persevering, tenacious, restless, assertive, determined r = 0.33, p = 0.20); Amicability (positive loadings: sociable, friendly r = 0.24, p = 0.36); Reservedness (positive loadings: nosey r = 0.42, p = 0.09)].

### Correspondence between behavioural analyses and questionnaire-based traits

To assess if the observed behaviour of the puppy could be associated with factor emerging from the personality questionnaire, a set of bivariate correlations was carried out (Table 6, Bonferroni correction for multiple comparisons).

**Table 6:** Pearson’ correlation coefficient and significance levels used to assess the correspondence between the behaviour factors identified by the hierarchical cluster analysis (rows) and personality factors emerging from the questionnaire-based analyses (columns).

	Extraversion	Neuroticism	Persistence	Amicability	Reserved
Exuberant attitude	0.565**	-0.380**	0.433**	0.492**	-0.400**
Cautious attitude	-0.389**	0.423**	-0.412**	-0.267*	0.119
Relaxed attitude	0.146	-0.318**	-0.071	0.164	-0.290*
Social Interaction	0.235	-0.087	0.203*	0.445**	-0.133
Playful interaction	0.204*	-0.262*	0.445**	0.106	-0.170
Non-stimuli related behaviour	-0.472**	0.534**	-0.468**	-0.450**	0.607**

*/** Identifies the significant effects which can be maintained after Bonferroni correction (acceptable values p<0.05 and p<0.01, respectively)

The exuberant attitude cluster, i.e. running around the arena with exuberant approach and interactions to objects and people, was positively correlated to Extraversion (r = 0.565, p<0.01), Persistence (r = 0.433, p<0.01) and Amicability (r = 0.492, p<0.01), while negatively correlated to Neuroticism (r = -0.380, p<0.01) and Reservedness (r = -0.400, p<0.01). The cautious attitude cluster, i.e. looking at people/object from a distance and approaching/interacting with the stimuli with a cautious posture, was positively correlated to Neuroticism (r = 0.423, p<0.01) and negatively to Extraversion (r = -0.389, p<0.01) and Persistence (r = -0.412, p<0.01). The relaxed attitude cluster, i.e. walking around the arena with a positive/neutral approach/interaction to the stimuli, showed a negative relation to Neuroticism (r = -0.318, p<0.01) and Reservedness (r = -0.290, p<0.05). Social interaction cluster correlated positively with Amicability (r = 0.445, p<0.01) and playful interaction was positively correlated to Persistence (r = 0.445, p<0.01). Non-stimuli related behaviour was strongly positively correlated to Neuroticism (r = 0.534, p<0.01) and Reservedness (r = 0.607, p<0.01), whilst negatively associated to Extraversion (r = -0.472, p<0.01), Persistence (r = -0.468, p<0.01) and Amicability (r = -0.450, p<0.01).

To summarise, the Extroversion trait from the questionnaire associated positively with the behavioural cluster subgroup expressing an energetic approach to the social and non-social stimuli in the test environment (e.g. fast gait, exuberant approach/interaction, playful interaction) and negatively with those expressing a more cautious approach (e.g. look stimulus, cautious approach/interaction) or a lack of interest in the stimuli (i.e. non-stimuli related behaviours). Interestingly, the analysis identified a very similar pattern of behaviours for the trait Persistence (Table 6), which supports the strong positive correlation between these two traits.

Conversely, the Neuroticism trait of our questionnaire was positively associated with cautious attitude (i.e. look at stimulus, cautious approach/interaction) and the lack of engagement with the stimuli, hence it describes puppies who either explored the stimuli but in a cautious manner, or largely did not interact with these but rather spent time sitting/lying or sniffing around and/or interacting with other elements of the environment (e.g. grass, leaves etc.) Furthermore, this trait correlated negatively with both behavioural dimensions describing positive interactions with the stimuli (exuberant and relaxed attitude) and playful interactions.

The Amicability trait was correlated positively to the social interaction dimension, confirming its description, but it also correlated positively to exuberant attitude, and negatively to cautious attitude and non-stimuli related behaviour. Indeed both exuberant and cautious attitude included respectively a positive and friendly or a more cautious interaction with the person, hence, these correlations are to be expected. The negative correlation with non-stimuli related behaviour is also to be expected since this largely described a puppy that mostly did not interact with the stimuli including the person present. Finally, Reservedness, reflected a puppy who largely chose not to interact with the stimuli (animate and inanimate), since it correlated significantly with non-stimuli related behaviours (e.g. sitting/lying, sniffing around), but negatively with both dimension describing positive interactions with the stimuli (exuberant and relaxed attitude).

## Discussion

The aim of this study was two-fold. First, to assess whether individual personality traits could be detected in puppies as early as 2-months of age applying tools that are largely used to assess personality in dogs (i.e. an adjective based questionnaire and a behavioural coding method). Second, to investigate the correspondence between these two methods in defining behavioural patterns. Overall results from both the adjective based questionnaire and the behavioural analysis suggest that at 2-months of age, when exposed to both social and non-social stimuli in an open-field test, puppies already show specific behavioural patterns which can be identified as relating to personality traits. Both methods proved to be reliable tools for the assessment of personality traits in as much as the inter-observer reliability was high in both cases, confirming previous findings [32] and a good correspondence in personality traits emerged between the two independent measures used. A further test of the strength of specific personality traits is represented by potential consistency found over time in the subset of puppies re-tested 2 months later.

A number of personality traits emerged. Based on the behavioural analyses, a first clear cluster which expressed the puppies’ ‘style’ of interacting with both social and non-social stimuli emerged describing an Exuberant versus Cautious attitude. From the questionnaire the first two factors (Extraversion and Neuroticism), largely identified the same pups falling in the Exuberant vs. Cautious factor dimensions, and indeed the high correspondence between these traits confirmed that there is a consistent behavioural pattern in the subjects tested. Overall these traits describe how pups interact with the stimuli in the test, whether they keep a distance and look at them from afar or whether they choose to explore them in an exuberant manner. In many respects this can be equated to the shyness-boldness dimension identified by other studies on dogs [17,27]. The shyness-boldness trait is a dimension, which has been described for many animal species [55], and from the evolutionary perspective it appears to be adaptive since it allows animals to cope with fluctuating environmental conditions [56]. In dogs it is likely that this trait has been maintained from the wolf-ancestor, although it is an open question to what extent wolves and dogs may differ in their representation along this continuum. Regardless, it is considered to be one of the more stable personality traits in dogs [27] and this seems to be confirmed also in the current study, by the fact that it was the only trait showing also temporal consistency in the test-retest reliability analyses.

A separate cluster, although closely linked to the exuberant/cautious attitude one, emerged for sociability (termed ‘Social interaction’) expressed by the time spent interacting with the people in the arena in a friendly manner. A very similar dimension emerged from the questionnaire (termed ‘Amicability’). Looking at the correspondence between the two methods it emerges that Amicability positively correlates with Social interaction and with the Exuberant attitude from the behavioural analysis, thereby confirming the link between these traits. The ‘sociability’ trait has been identified by a number of prior studies on puppies, suggesting it is one of the most easily identified [34]. However, in some studies the sociability trait emerged as embedded within the more general shy-boldness axis [27]. Results from our study are mixed in that it is closely associated with the exuberant attitude although it emerged as a separate factor. Surprisingly, consistency was not confirmed by our test-retest. Given that the puppies were tested twice in the same environment, with no major changes (e.g. adoption, change of home) affecting their social life experiences, we would have expected higher correlation estimates for this trait. Whether this was due to our experimental constraints (i.e. small sample size) or to the particular sensitivity of this trait during development is unclear and needs to be investigated further. Contrasting evidence emerges from the literature. The sociability trait has been reported to be moderately stable over time in adult dogs [57], however, studies on puppies are far fewer. Scott and Fuller [58] reported that social investigation and attraction toward humans remains fairly consistent after 7 weeks of age (p 137). However, a recent review found little consistency over time for this trait in puppies [3]. This discrepancy may depend on several factors: test-retest interval plays an important role in detecting consistency of personality traits (the larger the test interval the smaller the strength of consistency) [3] and age at testing is also known to affect consistency, with several studies showing that testing puppies at less than 12 weeks of age is not predictive of future behaviour [10,18,59].

Playfulness remained as a distinct cluster characterized by those pups spending their time in playful interactions whether with the inanimate stimuli or the experimenter. This factor correlated the most with the Persistence trait emerging from the questionnaire, probably because it described those pups who were persistent in their attempts to play with the experimenter (who was instructed to largely not respond to these attempts), and who persevered in playing with a specific stimuli (e.g. toy) somewhat to the exclusion of all else. Interestingly, the playful trait showed a positive correlation with Extraversion and a negative one with Neuroticism. In a seminal work, Svartberg [60] showed how the selective pressure on dog breeds is still in progress shaping, and significantly affecting the personality of modern breeds and breed lines. Importantly, he reported that popular modern breeds have higher sociability and playfulness scores than both less popular breeds and breeds used in shows, highlighting that both these aspects of a dogs’ behaviour are potentially very salient for pet owners. Indeed, Svartberg’s work suggest that playfulness is a stable trait and could indeed be defined a personality dimension in dogs, confirmed by its stability over time in adult dogs (correlation estimates 0.76–0.89) [25]. However, recent reviews on dog personality [3,34] have not identified playfulness as a trait and, in a recent study, no consistency over time from puppy- to adult-hood was found [59]. Never the less, studies on puppies are still few, hence given the relevance of this behaviour for pet owners, future studies should indeed aim at investigating this aspect of dog’s behaviour further.

The final behavioural cluster emerging, was labelled ‘non-stimuli related behaviour’. It identified puppies that spent most of their time not interacting with the stimuli presented, but rather displayed either passive behaviours (sitting and lying down), or they moved around sniffing the environment but without showing expressions of fear or anxiety. This cluster showed the highest correspondence with the Reservedness trait from our questionnaire, effectively describing a pup which was the opposite of nosey/curious and rather quiet. Results from both measures taken together then, describe those pups that mostly ‘do their own thing’, are not so interested in exploring the stimuli, but do not show great anxiety related to these.

Overall, the correspondence scores emerging from the current study are comparable to other studies with adult dogs in which correspondence between subjective and behavioural methods were found [29,30,32,61]. Indeed, the factors emerging from the personality questionnaire and the behaviours largely showed a coherent picture with a good correspondence between the two methods of analyses. The adjective-based questionnaire, despite not being specifically designed for the current study, showed remarkably similar dimensions to its previous use with adult pet dogs. It easily identified the major dimensions of Extraversion, and Neuroticism and although with a curtailed set of adjectives it also largely allowed the amicability dimension to emerge unchanged compared to the adult study. The larger differences emerged in the Persistence dimension which only partly reflected Ley et al.’s Self-assuredness/Motivation dimension, and the Reservedness dimension which was largely novel. The fact that the adult and puppy dimensions emerging are not identical may be due to the different use of the questionnaire i.e. in an open field test for puppies, or in a pet every day situation for adult dogs, and/or to the fact that the questionnaire used for this study was adapted, i.e. some terms were omitted because not suitable. This could have affected the reduction analysis and factor loadings. However, it may also be that dimensions at this young age are somewhat different. Interestingly, this phenomenon has been reported in developmental psychology where age-specific personality dimensions, independent of the Big Five in adults were reported in adolescents [62]. Future research will be needed to assess whether a similar pattern is occurring also in dogs.

A number of limitations in the current study need to be kept in mind. Jones and Gosling [63] suggested that an important aspect of personality assessment was the consistent emergence of similar traits in different context. Indeed, in a recent study on adult dogs [61], authors looked at the correspondence between dogs’ personality traits as assessed by owner questionnaire and the analyses carried out by researchers on the dogs’ behaviour during a temperament test. In our own study, although similarly to the study with adult dogs we sought to assess the correspondence between a questionnaire-based and behavioural-based analysis, the context remained the same: the open-field test. Future developments of this study would include testing the two different methods to assess puppies’ personality in various experimental contexts (e.g. a playful session with a stranger, and or potential conflict over a food source).

The assessment of the trait’s consistency over time, which is necessary for a factor to be considered a stable personality trait [3] is another limit of the present study. Considering that we were able to re-test only 18 puppies, the conclusion as to the stability over time of the different personality factors identified in the current study are potentially premature and would need confirmation with a larger sample size. The subsample of puppies we were able to test did not undergo any specific selection, in that some were pups that the breeder decided to keep as future breeding stock (which could have been chosen on both morphological or behavioural traits) whilst others were pups that were not yet sold/ given away. Even though there was no systematic bias in the choice of pups the sample size is very small. Nevertheless, consistency over time emerging for the two main behavioural aspects (exuberant and cautious attitude) and questionnaire based personality traits (extroversion and neuroticism) that were also highly correlated with each other, suggest that these factors may form the basis of a dogs’ developing personality. Thus, although further studies with puppies are needed to confirm results on the consistency of personality traits over time, current results on the factors showing correlation between the two time periods are comparable to those reported in previous studies for adult dogs [18,23,31].

Finally, since the aim of the current study was an evaluation of two methods for assessing personality traits in puppies, we sought to maximize the variability of puppies represented in the sample, thereby including subjects from 21 different breeds differing in size and belonging to different breed groups. Nevertheless, some breeds were represented more than others, and breed (at least in adult dogs) has been shown to affect personality [60,64]. Although potential breed differences in the representation of personality traits does not alter current results in terms of the evaluation of the two methods adopted, it is possible that with a different sample of puppies from a different set of breeds, other personality traits would emerge, that were not observed in the current study. Considering breed difference in personality traits have both theoretical (e.g. the effect of selection) and applied (e.g. selection for specific ‘working’ purposes) future research on this aspect would be particularly welcome.

In summary, despite the use of two very different tools, a more easy-to-apply adjective based questionnaire (scored on a 5-point scale) and a more complex and demanding (in terms of time and experience) tool as the behavioural coding (recording frequency and duration of behaviour) results were rather consistent and showed good correlations. The consistent identification of two main ‘types’ of puppies was easily detectable while scoring the puppies in the open-field test. Following the descriptions provided in this paper of Extrovert (Exuberant) or Neurotic (Cautious) puppies, breeders could easily profile the puppies in their litters. As mentioned above, this does not ensure the stability of those traits in dogs’ adulthood never the less it gives a good indication of the present attitude of a pup and could be of help when selecting the most appropriate future family.

## Supporting Information

S1 Information Questionnaire selection method. Description of the questionnaire selection procedure.
(DOCX)
