# Supplementary material for: Does Subjective Rating Reflect Behavioural Coding? Personality in 2 Month-Old Dog Puppies: An Open-Field Test and Adjective-Based Questionnaire
Source: PLoS One. 2016 Mar 15;11(3):e0149831. doi: 10.1371/journal.pone.0149831 (PMC4792536; doi:10.1371/journal.pone.0149831)
Supplement: S1 Information — Description of the questionnaire selection procedure. (DOCX) [file pone.0149831.s001.docx]

**S1 Supporting information**

**Questionnaire Selection Method**

An initial four potential adjective based questionnaires were taken into consideration (the canine-BFI, Gosling et al. 2003; the MCPQ/MCPQ-R, Ley et al. 2007, 2008; Kubinyi et al. 2009; Free-profiling method Walker et al. 2010). Two of the studies (Ley et al. 2007,2008, Walker et al. 2010), were based on a list of adjectives either determined a priori (Ley et al. 2007) or generated by subjects themselves (Walker et al. 2010), whereas the remaining two questionnaires were based on questions addressed to the owner which however contained specific adjective (e.g. ‘is cool-headed even in stressful situations’ or ‘is emotionally balanced’ Kubinyi et al. 2009; ‘shows a lot of enthusiasm’ or ‘can be cold and aloof’ Gosling et al. 2003). On closer inspection Walker et al.’s was excluded because although the free-profiling method has been used successfully with dogs, the factors emerging from this study contained many adjectives which were repeated in more than one factor, which is problematic since it questions the possibility of analysing the internal consistency of the questionnaire, and direct correspondence with the factors emerging from the behavioural coding. Furthermore, the Kubinyi et al. (2009) study also used questions which although adjective based, on many occasions described everyday situations (e.g. is ingenious, inventive when seeks hidden food or toy) or interactions with conspecifics (e.g. is shy when interacting with conspecifics), situations which could not apply in our setting.

From the Gosling et al. study in which adjectives were presented in a question format, we extrapolated the single words and made a list. This resulted in a list of 47 adjectives from the Gosling et al. (2003) study and a list of (41) adjectives used in the Ley et al. 2007 study. Both lists was given to 5 observers (authors of the current paper, two of which had already coded the pups’ behaviour and 3 who had not) who were asked to rate 15 videos of puppies in the open field test on all adjectives appearing in each list on a 5 point scale (1 does not describe this pup at all – 5 really describes this pup). Furthermore, the 5 observers were asked to indicate which terms they thought could not be applied to pups on the basis of the open-field test. Adjectives considered to be inappropriate by 3 or more of the 5 observers were excluded. Cronbach’s alpha coefficient was used to evaluate the inter-observer reliability between the 5 observers based on the remaining adjectives.

In both cases adjectives relating to the ‘trainability’ (Ley et al. 2007) and ‘openness/intelligence’ dimension were mostly considered to be impossible to evaluate based on the open field test. Hence of the 7 adjectives weighing on this dimension in the Ley study only 1 (i.e. attentive) was deemed appropriate to use in our study; whereas only 3 of the 8 adjectives used in the Gosling study could be maintained (i.e. curious, thoughtful, appreciative of sensory experiences). Both studies revealed an extraversion and a neuroticism dimension, and adjectives referring to these two traits were almost all considered to be applicable to the current study (extraversion: 7/8 adjectives in Gosling et al.; 10/10 adjectives in Ley et al. 2007; neuroticism: 7/8 adjectives in Gosling et al.; 5/6 adjectives in Ley et al. 2007). Both studies also revealed a dimension referring to the dog’s sociability, however a different emphasis emerged in the two studies. In Gosling et al. the dimension ‘agreeableness’ includes aspects, which refer to generous interaction towards another (e.g. forgiving, trusting, sensitive to others, cooperative) whereas in the Ley study, the ‘amicability’ dimension refers to a more general characteristic (e.g. friendly, sociable, gentle etc.). Predictably, whereas all the eight adjectives weighing on the ‘amicability’ dimension were considered to be applicable to our current setting by the observers, only 3 of the 9 adjectives referring to ‘agreeableness’ were. Finally, the Ley et al. study identified an extra dimension not present in the Gosling study: ‘self-assuredness’ identified by 10 adjectives most of which (6) were considered to be appropriate also for the current study. Mean inter-observer reliability was 0.79 for both lists of adjectives used.

Considering that for most dimensions a greater number of adjectives were considered appropriate when assessing the open field tests using Ley et al.’s questionnaire, and an extra dimension would be maintained with this questionnaire we adopted a modified version of this questionnaire. Considering only one adjective referring to the dimension ‘trainability’ was maintained, this dimension was dropped completely from our questionnaire. The four adjectives referring to self-assuredness, which were thought not to be applicable (i.e. thorough, proud, opportunistic, dominant) and the adjective submissive (included in the neuroticism category) were also excluded. Finally, the adjective ‘unaggressive’ and ‘non-aggressive’ were removed because all puppies received the same scoring on these elements (i.e. all were non aggressive), suggesting that at this age, this variable is not particularly discerning. In all other respects the list of adjectives adopted was identical to that used by Ley et al. (2007) (see Table 2 in main text).
